# NTRK Fusions, from the Diagnostic Algorithm to Innovative Treatment in the Era of Precision Medicine

**DOI:** 10.3390/ijms21103718

**Published:** 2020-05-25

**Authors:** Federica Zito Marino, Francesca Pagliuca, Andrea Ronchi, Immacolata Cozzolino, Marco Montella, Massimiliano Berretta, Maria Elena Errico, Vittoria Donofrio, Roberto Bianco, Renato Franco

**Affiliations:** 1 Pathology Unit, Department of Mental and Physic Health and Preventive Medicine, University of Campania ‘Luigi Vanvitelli’ Complesso di Santa Patrizia, Via Luciano Armanni, 580131 Naples, Italy; federica.zitomarino@unicampania.it (F.Z.M.); frances.pagliuca@gmail.com (F.P.); andrea.ronchi@unicampania.it (A.R.); coimma73@gmail.com (I.C.); marco.montella@unicampania.it (M.M.); 2 Department of Medical Oncology, Istituto Nazionale Tumori (IRCCS), Centro di Riferimento Oncologico di Aviano, Via Franco Gallini 2, 33081 Aviano (PN) Italy; berrettama@gmail.com; 3 Pathology Unit Department of Pathology, Santobono-Pausilipon Children’s Hospital, Via Posillipo, 80123 Naples, Italy; mariaelenaerrico@virgilio.it (M.E.E.); vittoriadono@gmail.com (V.D.); 4 Oncology Unit, Department of Clinical Medicine and Surgery, University of Naples “Federico II”, Via Pansini, 80131 Naples, Italy; robianco@unina.it

**Keywords:** neurotrophic tyrosine receptor kinase (NTRK) fusions, NTRK1, NTRK2, NTRK3, ETV6-NTRK3, NGS, FISH, Pan-TRK IHC, tumor biobanks

## Abstract

In the era of precision medicine, the identification of several predictive biomarkers and the development of innovative therapies have dramatically increased the request of tests to identify specific targets on cytological or histological samples, revolutionizing the management of the tumoral biomaterials. The Food and Drug Administration (FDA) has recently approved a selective neurotrophic tyrosine receptor kinase (NTRK) inhibitor, larotrectinib. Contemporarily, the development of multi-kinase inhibitors with activity in tumors carrying TRK fusions is ongoing. Chromosomal translocations involving the NTRK1, NTRK2, and NTRK3 genes result in constitutive activation and aberrant expression of TRK kinases in numerous cancer types. In this context, the identification of tumors harboring TRK fusions is crucial. Several methods of detection are currently available. We revise the advantages and disadvantages of different techniques used for identifying TRK alterations, including immunohistochemistry, fluorescence in situ hybridization, reverse transcriptase polymerase chain reaction, and next generation sequencing-based approaches. Finally, we propose a diagnostic algorithm based on histology and the relative frequency of TRK fusions in each specific tumor, considering also the economic feasibility in the clinical practice.

## 1. Introduction

In recent years, the identification of a large number of specific actionable oncogenic mutations, such as gene-activating point mutations and chromosomal abnormalities involving critical oncogenes, has dramatically changed the natural history of some solid tumors, opening the way for a new medical science, the so-called precision medicine [1].

The precision medicine extends its branches into the fields of both cancer research and care, having the purpose of developing prevention and treatment strategies. Indeed, the identification of valid potentially targetable strategies represents the current goals of cancer research, hopefully applicable in the clinical settings [2]. In this field, a new promising predictive biomarker is represented by neurotrophic tropomyosin receptor kinase (NTRK), as selective inhibitors of the constitutively active NTRK fusion proteins have been developed and could be introduced in the treatment of solid tumors carrying NTRK chromosomal aberrations [3,4]. In the history of NTRK identification and development as a clinical biomarker, the tumoral tissue played a core role for both the cancer research and subsequent clinical application. Thus, the need of NTRK rearrangement identification in tumoral tissue favored the development of diagnostic tools applicable through both classical ‘morphological’ methodologies, such as immunohistochemistry (IHC) and fluorescent in situ hybridization (FISH) and high-throughput molecular characterization of tumor tissues, such as next generation sequences (NGSs) [1]. In NTRK identification, such as in the field of precision medicine, the classical role of the pathology unit in both the cancer research and clinical setting has deeply changed, on the one hand because of more requests of competences in the field of the molecular pathology and on the other hand for the need of the correct identification and quantization of the tumoral areas [1]. For instance, more than 90% of developed oncological drugs do not enter the clinical application, because of the failure of the specific trials, potentially due also to the inappropriate choice of the tumoral tissues [5]. In this view, the wise use of biomarkers and the qualified selection of tumoral tissues will be the basis of minimizing the risk of clinical trials’ failure. In addition, the hard and expensive process of developing cancer biomarkers to use in the clinical field requires the multidisciplinary collaboration of academic researchers, hospital clinicians, pathologists, molecular biologists, technicians, biostatisticians, regulators, and pharmaceutical companies. Finally, the identification of validated biomarkers on tumoral tissues in the clinical practice for a specific therapeutic approach through morphological or molecular tools pre-requires the correct identification and quantization of the tumoral areas in adequately collected and processed tissues, according to the standard operative procedures in use in the pathology unit lab, including different professional figures, such as pathologists, molecular biologists, molecular oncologists, and technicians [1].

In this review, we discuss the optimal management of biomaterials and the technological approaches to detect NTRK rearrangements. In a multidisciplinary vision of oncologic patients, an expert opinion regarding the treatment of a TRK fusion-associated tumor is reported. Finally, we propose a differential diagnostic algorithm based on the relative frequency of NTRK rearrangements, high or low, in each specific tumor.

## 2. NTRK Genes: Structure, Function, and Oncogenic Potential

The NTRK1, 2, and 3 genes encode a family of tyrosine kinase receptors with an active role in neural development. All rearrangements cause constitutive activation of these proteins [4,6,7]. NTRK rearrangements have been reported in a series of solid and hematological tumors, with variable frequencies (Table 1). These recent discoveries raise diagnostic and therapeutic challenges.

TRK (tropomyosine receptor kinase) receptors are a family of neurotrophin-binding transmembrane proteins with a role in neuronal development and differentiation, physiologically expressed during embryonic development and in the adult nervous system [8,9]. They are encoded by three different NTRK genes, namely *NTRK1*, located on chromosome 1q21-q22, *NTRK2*, on chromosome 9q22.1, and *NTRK3* on chromosome 15q25. The corresponding receptors TrkA, TrKB, and TrkC have a similar structure, each showing a higher affinity for a specific neurotrophin and activating different intracellular pathways. In particular, TrkA binds to NGF (nerve growth factor) and TrkB binds to BDGF (brain-derived growth factor), both leading to the activation of the MAPK/RAS/ERK, PLC-γ (phospholipase C-gamma), and PI3K (phosphatidylinositol 3-kinase) pathways [10]. They act on neuronal proliferation, differentiation, and survival. On the other hand, TrkC, whose ligand is NTF-3, employs PI3/AKT as a downstream effector and plays a major role in contrasting neuronal apoptosis. Actually, Trk receptor-mediated signaling also exerts multiple crucial effects on neuronal function and plasticity, including axon, dendrite, and synapse formation.

The first evidence of NTRK genes’ role in cancer development dates back to more than 30 years ago, when NTRK fusions were described in colorectal and thyroid tumors [11,12]. Since then, NTRK gene aberrations have been described in multiple adult and pediatric neoplasms. Gene fusions represent the best understood mean of oncogenic NTRK activation. In fact, single nucleotide or splice variants and gene copy number alterations are also sporadically observed, but their clinical significance is still poorly characterized. NTRK fusion genes are the result of intra- or inter-chromosomal rearrangements, the former being the most common event type for *NTRK1*. Several fusion partners for NTRK genes have been identified so far. In all cases, the hybrid genes retain the 3′ region of NTRK, where the thyrosine kinase domain is found, juxtaposed to the 5′ region of the fusion partner, which is usually a ubiquitously expressed protein containing oligodimerization domains. The result is the ligand-independent activation of the thyrosine kinase domain in the aberrantly expressed fusion oncogene [13].

NTRK oncogenic fusions can be encountered in two main different scenarios: One consists of rare tumors in which NTRK fusions are found at very high frequencies, as dominant oncogenes (infantile fibrosarcoma, mammary secretory carcinoma, mammary-analogue secretory carcinoma of salivary glands, congenital mesoblastic nephroma) while the other comprises common tumors in which NTRK fusions are identified at low frequencies, including both solid and hematological malignancies [4].

## 3. Tumors Harboring NTRK Gene Aberrations

**Figure 1:**
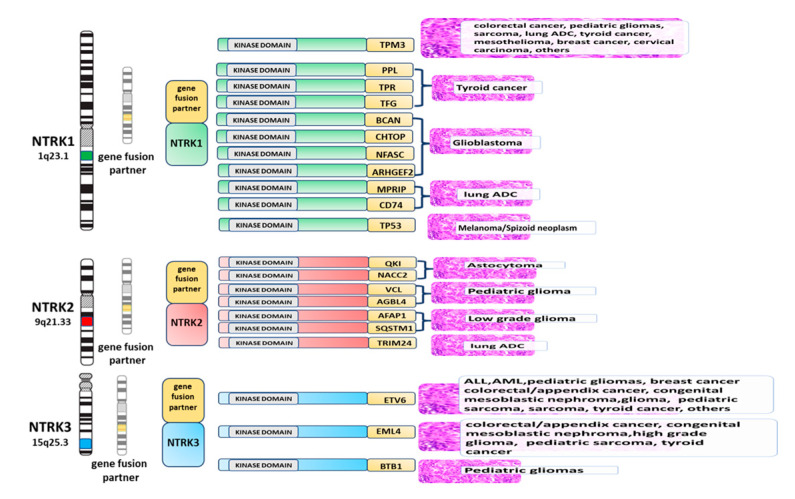
NTRK gene fusions in cancers. A schematic diagram of the known NTRK gene fusion partners is provided. Partners of NTRK1, NTRK2, and NTRK3 are stratified according to the cancer type where they are most frequent.

As previously mentioned, *NTRK* aberrations are rare in most common malignancies, being found at a frequency of <5%, mostly ranging between 0.1% and 2% according to the tumor type. Nevertheless, a few rare histotypes are highly enriched for *NTRK* alterations, specifically chromosomal translocations (Figure 1). They include infantile congenital fibrosarcoma and congenital mesoblastic nephroma (cellular and mixed subtypes), pediatric tumors that can have an aggressive course, as well as secretory carcinomas of the breast and salivary gland. All these tumors share a recurrent *ETV6-NTRK3* translocation, which is found in >75% of cases (up to 90% in some series). Originally discovered on infantile fibrosarcoma, the detection of *ETV6-NTRK3* has a well-established role in differentiating this entity from other pediatric spindle cell tumors [14]. Along with its diagnostic utility, the presence of this translocation has recently led the way to the successful use of NTRK inhibitors in the neoadjuvant and adjuvant setting for young fibrosarcoma patients [15]. Similarly, an extraordinary response to larotrectinib, a selective inhibitor of Trk receptors, has been reported in one patient with refractory fusion-positive secretory breast carcinoma, suggesting that targeted therapy could be an effective alternative to chemotherapy in this unusual triple-negative neoplasm accounting for only 0.15% of all breast carcinomas [16,17,18].

However, it is important to remember that, apart from ETV6, other fusion partners for *NTRK3* or *NTRK1* fusions have also been described in this group of tumors [19,20,21]. On the other hand, a subset of mammary-analogue secretory carcinomas of the salivary gland are seen to harbor *ETV6* translocations not involving NTRK genes and correlating with a less favorable behavior [22,23].

*ETV6-NTRK3* fusion also occurs quite commonly in a subset of radiation-associated and pediatric papillary thyroid carcinomas (PTCs), representing the prevalent gene rearrangement in this setting after RET-PTC, while it is rare in the sporadic adult population [24,25]. Some authors have attempted to define the clinical and histopathological features of *ETV6-NTRK3* translocated PTCs, highlighting a predominantly follicular or mixed follicular and papillary growth pattern with frequent oncocytic and clear cell change, deceptively bland nuclear features, and an increased prevalence of lymph node metastases. A background of chronic lymphocytic thyroiditis was also noted [26,27].

Apart from *ETV6-NTRK3* translocations, multiple fusions involving *NTRK1* have been identified in PTC [22]. *TPM3* (1q22-23), *TPR* (1q25), and *TGF* (3q11-12) are the most common fusion partners. The frequency of such translocations in sporadic PTC is around 12% with geographic variations across different populations. NTRK1 aberrations correlate with a younger patient age at diagnosis, locally advanced disease, and a less favorable outcome. No association with a specific tumor subtype has been reported so far [28,29].

*NTRK1* fusions have been described as the most common kinase fusions, together with those involving *ROS1*, in the entire biologic spectrum of spitzoid neoplasms, including benign Spitz nevi, atypical Spitz tumors, and spitzoid melanomas. *NTRK1* fusions seem to occur in a mutually exclusive pattern with other kinase fusions (*ROS1*, *ALK1*, *RET*, *BRAF*) [30]. In addition, more recently, *NTRK1*, *NTRK2*, and *NTRK3* kinase fusions have been detected in a very small proportion (<1%) of metastasizing non-spitzoid melanomas of adults, suggesting the opportunity of Trk inhibition therapy for this group of patients [31].

Among the ‘big killer’ cancers, NTRK rearrangements are fairly uncommon. First identified in 1986, *TMP3-NTRK1* fusion is a rare but recurring event (0.5%) in colorectal carcinoma (CRC) [11,32].

Of notice, a novel *LMNA-TNRK1* fusion has lately been identified in one metastatic CRC patient refractory to standard therapy [33]. The patient was successfully treated with the multi-kinase inhibitor entrectinib, achieving an objective partial response.

Less than 5% of non-small cell lung cancer (NSCLC) harbors NTRK fusions, which are sporadically found in a non-specific clinical or histopathological background [34,35,36].

Although infrequent, evidence exists that tumors harboring *NTRK1* rearrangements, including those with central nervous system metastases, could show good responses to entrectinib [37].

On the other hand, contrasting results have been reported regarding *NTRK2*’s role in NSCLC. In a recent work on comprehensive bioinformatic analysis of different datasets, it has been hypothesized that NTRK2 may act as an oncosuppressor in lung adenocarcinoma. Its expression levels were markedly decreased in tumor tissue and its downregulation and higher levels of methylation were associated with worse overall survival and relapse-free survival [38]. Other works suggests that BDGF-Trk2 signaling could promote cellular plasticity and an invasive migratory phenotype in squamous NSCLC and therefore TrkB could be an actionable target in lung squamous cell carcinoma [39,40].

Other tumors occasionally reported to harbor NTRK aberrations include astrocytomas, glioblastomas, intrahepatic cholangiocarcinomas, head and neck squamous cell carcinomas, and chronic myeloid leukemia.

## 4. The Choice of Biomaterials to Detect NTRK Fusions

The continuous improvement of our understanding of the tumor biomolecular landscape is determining the development of increasingly more numerous pharmacological molecules for targeted therapy. Consequently, many biomarkers have to be tested on biological samples to select the patients who can benefit from personalized therapies. A high number of predictive biomarkers have to be added to the immunohistochemical and molecular markers performed for diagnostic purposes. So, the way the pathologists manage the biological material is rapidly changing, mainly in the case of small bioptic samples. Actually, the choice of the invasive technique to obtain the biomaterial is not only linked to classical considerations (cost, safety, availability on the territory, etc.) but also to the chance of testing all the necessary biomarkers on that type of biomaterial. The evaluation of NTRK status requires the execution of IHC and FISH at least. The experience about NTRK testing is still limited, and all the studies have been performed on FFPE (formalin-fixed and paraffin-embedded) tissue [41]. General recommendations, for small biopsies, include performing upfront multiple sections for diagnostic and predictive tests, and minimizing the immunohistochemical panel for diagnostic purposes, in order to limit the consumption of paraffin blocks [42]. As regards cytological samples, fine needle aspiration cytology (FNAC) is widely used for the diagnosis of many neoplasms, and cytological samples may represent the only available biomaterial in some circumstances. Cytological material is generally excluded from clinical studies and biomarkers are generally tested on histological samples. In this setting, the realization of a cell block obtained from FFPE cytological material could be fundamental to test the NTRK molecular status, although currently, there is no evidence that the test cannot be performed on alcohol-fixed direct smears. Certainly, the advantage of using a cell block compared to conventional smears is represented by the possibility of obtaining more sections, compared to the more limited number of direct smears. However, it is now known that stained diagnostic direct smears can be subsequently sacrificed by scraping the cells from the surface to produce sufficient DNA for targeted NGS [42]. Therefore, although it is possible to use any cytological sample, the cytopathologist must carefully choose which available material (cell-block section, direct smears) must be destined for one technique over another. Nevertheless, the diagnostic performance of NTRK IHC and FISH tests on cytological specimens or NGS and perhaps the optimization of multiplexed approaches have to be evaluated in a large series.

## 5. Methods for the Detection of NTRK Fusions

NTRK fusions can be detected using different techniques, including IHC, FISH, RT-PCR, and both RNA-based and DNA-based NGS.

Each assay shows both advantages and disadvantages, and currently, a precise diagnostic algorithm has not yet been defined. The methods of choice for NTRK1/2/3 fusion gene detection should take into consideration the histology and clinical context.

### 5.1. Immunohistochemistry (IHC)

**Figure 2:**
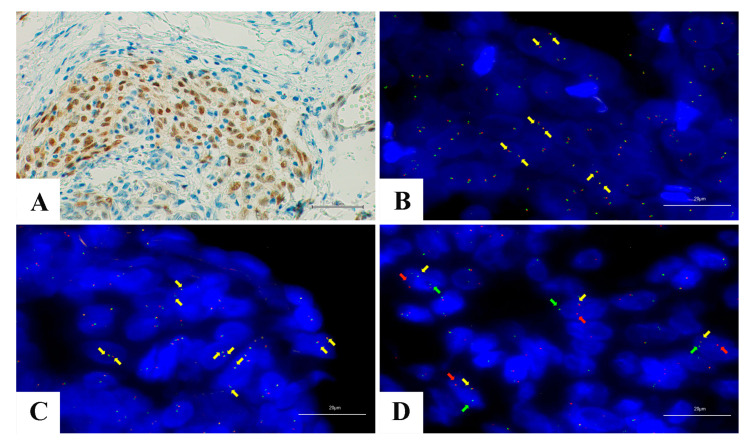
Infantile fibrosarcoma showing (**A**) NTRK-positive nuclear staining of the neoplastic cells (Immunohistochemistry VENTANA pan-TRK Assay (*EPR17341*), original magnification 400×); (**B**) Fluorescence in situ hybridization (FISH) showing an absence of NTRK1 rearrangement (ZytoLight SPEC NTRK1 Dual Color Break Apart Probe, original magnification 1000×): The native NTRK1 status (indicated by yellow arrows) shows fusion or closeness of the probes adjacent to the 3′ and 5′ ends of the gene, labeled, respectively, with red and green fluorophores; (**C**) FISH analysis showing an absence of NTRK2 rearrangement (ZytoLight^®^ SPEC NTRK2 Dual Color Break Apart Probe, original magnification 1000×): Native NTRK2 status (indicated by yellow arrows) shows fusion or closeness of the probes adjacent to the 3′ and 5′ ends of the gene, labeled, respectively, with red and green fluorophores; (**D**) FISH analysis showing the presence of NTRK3 rearrangement (ZytoLight^®^ SPEC NTRK3 Dual Color Break Apart Probe, original magnification 1000×): Rearranged NTRK3 is indicated by the presence of split 3′ (red arrows) and 5′ (green arrows) signals; yellow arrow shows single yellow native gene.

Although molecular tests are certainly the most sensitive and specific techniques to detect genetic aberrations, efforts are being made to investigate the potential role of IHC in screening cases harboring TRK mutations. IHC has numerous advantages, as it is inexpensive, safe, fast, widespread in all laboratories, and straightforward to implement and validate if compared with molecular tests. IHC could be a specific test for detecting TRK alterations in most tissues, as Trk proteins seem to be poorly expressed in normal adult tissues, being found only in smooth muscles, testes, and neural components [43,44,45]. As has already happened for other predictive biomarkers, the scientific community wonders if IHC will be able to identify cases deserving a genetic study for TRK aberrations. The topic is of recent interest and only a few studies have investigated the utility of IHC in this setting till now. Consequently, the currently available sensitivity and specificity values for TRK IHC derive from data on relatively small cohorts. Different IHC antibodies can be used, including antibodies targeting specific NTRK proteins (Trk-A or Trk-B), antibodies targeting common amino acid sequences present in all Trk proteins (pan-Trk antibodies), and antibody cocktails [46,47,48]. The most well-studied clone is the pan-Trk antibody EPR17341 (Abcam and Roche/Ventana), binding a homologous region of Trk-A, Trk-B, and Trk-C near the C-terminus. Although the staining intensity may be variable, IHC-positive cases showed a diffuse positivity in neoplastic cells in most studies. However, some authors proposed that neoplasm with at least 1% of positive neoplastic cells should be considered as positive [49]. Taking into account the expression of Trk proteins in normal human adult tissue, testis tissue, submucosal colonic ganglia, and nervous tissue may be used as positive control tissues in IHC tests. The staining pattern in neoplastic cells may be divergent compared to the physiological membrane localization of native Trk, depending on the localization of the fusion partner [49]. The first study about the utility of IHC as a screening test for NTRK fusions was published in 2017 by Hechtman et al. [48]. The authors tested the expression of Trk proteins in a heterogeneous group of 23 neoplasms characterized by known *NTRK1*, *NTRK2*, or *NTRK3* rearrangements, including large bowel adenocarcinomas, gallbladder adenocarcinomas, glioblastomas, lung adenocarcinomas, secretory carcinomas of breast and salivary glands, melanomas, and sarcomas [48]. IHC resulted a highly efficient test to detect TRK-rearranged cases in this study, with a sensitivity of 95.2%, specificity of 100%, positive predictive value of 100%, and negative predictive value of 96% [48]. Interestingly, the immunohistochemical staining pattern appeared to correlate with the specific genetic rearrangement. Indeed, the authors detected a nuclear membrane immunostaining in neoplasms with NTRK1-LMNA fusions, nuclear staining in neoplasms with ETV6-NTRK3 fusions, and a cell membrane staining in neoplasms with TRAF2-NTRK2 fusions and in fusions involving TPM3/4 [48]. This finding is probably due to the cellular localization of the fused protein, which is more often a consequence of the normal localization of the Trk fusion partner. For example, lamin A/C (encoded by the *LMNA* gene) is a structural protein of the inner layer of the nuclear membrane, while ETV6 encodes a nuclear-located transcription factor. Further studies have subsequently tested the value of IHC as a diagnostic test for TRK-mutated tumors, with a similar high performance in terms of sensitivity and specificity [47,49,50]. Nevertheless, the most recent data suggest that the performance of IHC in detecting TRK-mutated cases is not uniform, depending on the type of neoplasm and type of genetic translocation. In 2019, Gatalica et al. tested IHC on 4136 samples, including 28 NTRK fusion-positive cases [49]. IHC was positive in 87.5% of the NTRK1-mutated cases, and 88.9% of the NTRK2-mutated cases, but only 54.5% of the NTRK3-mutated cases. Moreover, Solomon et al. tested Trk IHC on a heterogeneous group of 66 neoplasms positive for NTRK fusions, finding sensitivity for NTRK1 and NTRK2 fusions of 96% and 100%, respectively, and sensitivity for NTRK3 fusions of 79% [51]. Consequently, a caveat of IHC is actually the reduced sensitivity for neoplasms with NTRK3 fusions. On the other hand, the specificity of IHC tests seems to be dependent on the histotype, the highest specificity (100%) being observed in carcinomas of the colon, lung, thyroid, pancreas and biliary tract, and melanomas [52]. In contrast, a high rate of false positive expression was reported in some histotypes, and particularly in neoplasms with neural and smooth muscular differentiation, implying that IHC specificity is lower in mesenchymal tumors with muscular differentiation and in nervous system neoplasms [50]. In 2020, Solomon et al. showed a specificity of just 20.8% in gliomas, due to strong background staining in the neuropil [51]. A high rate of cytoplasmic false positive staining has also been reported in neuroendocrine neoplasms, breast carcinomas, and salivary gland tumors (other than secretory carcinomas) [51]. IHC is emerging as a screening tool in detecting neoplasms with TRK aberrations. An algorithm was recently proposed by the European Society for Medical Oncology (ESMO) to detect NTRK fusions, according to which a diffuse and strong cytoplasmic staining should be considered a surrogate of NTRK1/NTRK2 fusions and nuclear staining should be considered a surrogate of NTRK3 fusions (Figure 2A). On the other hand, weak cytoplasmic staining should be confirmed by molecular tests [47,53].

### 5.2. Fluorescence in Situ Hybridization (FISH)

Historically, FISH was the gold standard for the identification of other recurrent gene rearrangements, for example, those involving ALK, ROS1, and RET in NSCLC. FISH is a DNA-based assay that can detect oncogenic fusions using either fusion probes or break-apart probes. This method has several advantages, including high sensitivity to the detection of canonical breakpoints, the possibility to localize the target in the tumoral cells, the quick turnaround time and the availability in several laboratories [54,55].

NTRK1, 2, 3 fusions’ detection through FISH requires three separate assays using specific break-apart probes for each gene, unless a multiprobe assay is developed. The need for three different assays represents the major limitation for using FISH as a screening method for NTRK fusions, in relation to the costs and the time consumption.

Break-apart FISH probes targeting NTRK1, NTRK2, and NTRK3 provide information related to the rearrangement of the genes but not to the fusion partners (Figure 2B–D).

FISH represents the standard method to detect ETV6-NTRK3 in the tumors in which this translocation is common, such as infantile fibrosarcoma, congenital mesoblastic nephroma, and secretory carcinoma of the salivary gland or the breast [22,56,57].

FISH shows high sensitivity and specificity in the detection of chromosomal abnormalities; however, it has limits in distinguishing breakpoints involving non-canonical sites or intrachromosomal rearrangements, leading to false-negative results [19,21].

These limitations may especially impair the detection of NTRK1 fusions, since they are frequently determined by intrachromosomal events involving chromosome 1 [6,52]. LMNA-NTRK1 fusion, for example, is caused by an intrachromosomal deletion that is undetectable by FISH due to insufficient splitting of the signals [52].

FISH has adequate sensitivity and specificity for NTRK gene fusions’ identification. In the future, the implementation of multiprobes simultaneously targeting NTRK1, 2, and 3 could increase its use in the clinical practice.

### 5.3. Reverse Transcriptase Polymerase Chain Reaction (RT-PCR)

Reverse transcriptase polymerase chain reaction (RT-PCR) is an RNA-based method that detects the presence of known fusion transcripts. This assay can be performed relatively quickly and at low costs. RT-PCR is able to detect NTRK gene fusions using primers in the coding sequence of the 5′ fusion partner and the NTRK kinase domain. The main disadvantage of this technique includes the inability to determine novel fusion partners. Since several different fusion partners and breakpoints are involved in NTRK fusions, RT-PCR has limited applicability in the clinical routine [58].

Previous studies assessed the use of RT-PCR to detect NTRK fusions in several cancer types, including thyroid cancers, glioblastomas, congenital fibrosarcomas secretory, carcinoma of the salivary glands, and breast cancer [18,22,29,59,60,61,62,63].

RT-PCR assays have been applied preferentially for the identification of canonical ETV6-NTRK3 fusions in infantile fibrosarcoma, secretory breast cancer, and congenital mesoblastic nephroma [17,59,64,65].

### 5.4. Next-Generation Sequencing

Next-generation sequencing (NGS) represents a useful method to detect NTRK fusions with higher sensitivity and specificity compared with other assays [58]. The major advantage of NGS is that the status of multiple oncogenes can be simultaneously investigated using the same tumor sample. Moreover, the NGS approach allows the identification of novel NTRK fusions compared with other testing techniques [58].

NGS can also be applied to detect NTRK fusions through plasma-based cell-free DNA (cfDNA) testing. Plasma-based NGS NTRK fusion detection represents a clinically effective alternative in oncologic patients with advanced disease when a biopsy is not feasible [6]. Several different NGS-based approaches are currently available for gene fusion detection, including both RNA- and DNA-based assays [66].

### 5.5. DNA-Based Next-Generation Sequencing

The DNA-based NGS assay allows the analysis of genomic DNA to concurrently assess the somatic mutational status of many genes according to a panel of cancer-related genes or to whole exome and genome sequencing. A major advantage of NTRK fusion detection through DNA-based NGS testing is related to the simultaneous assessment of several genetic aberrations, such as point mutations, copy number variants, and tumor mutation burden [52]. DNA-based NGS represents a useful tool for monitoring patients harboring NTRK fusion-positive tumors treated with TRK inhibitor therapy that developed resistance mutations [53,67,68]. However, DNA-based NGS can fail to detect all types NTRK gene fusions, especially those involving NTRK2 and NTRK3 since these genes have several common fusion breakpoints within large intronic regions containing high numbers of repetitive elements, causing inadequate sequencing [66,69].

### 5.6. RNA-Based Next-Generation Sequencing

RNA-based sequencing presents some advantages compared to the DNA-based approach, namely the sequencing of mature mRNA, which is not affected by intron size or the presence of fusions involving multiple genes and exons simultaneously [66]. RNA-based NGS methods could be considered the gold standard to detect NTRK fusions. One of the main limitations associated with this method remains the RNA quality, since RNA is more labile than DNA and it is frequently degraded in FFPE tissue. Lately, new reagents have improved the efficiency of RNA library preparation [70]. Furthermore, platforms assessing in the same run both RNA and DNA libraries have recently been developed [52]. The NGS approach concurrently analyzing DNA and RNA could represent the next frontier in cancer for the identification of multiple biomarkers in a single assay, including NTRK fusions.

## 6. Diagnostic Algorithm to Detect NTRK Gene Fusions

**Figure 3:**
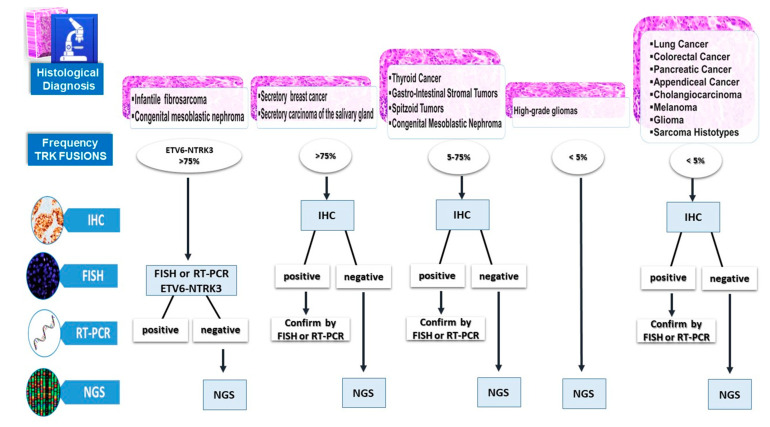
Different methodological approaches to NTRK rearrangements according to incidence-group tumors.

The fact that NTRK gene fusions typically occur in a mutually exclusive fashion with other oncogenic drivers and the clinical benefit derived from the treatment with selective TRK inhibitors underline the relevance of identifying patients who may benefit from effective and personalized therapies. We propose a diagnostic algorithm to select patients carrying NTRK fusions in relation to the histopathologic features of the tumor, the related frequency of TRK fusions, and the different available techniques (Figure 3). We suggest a stratification based on the histopathological diagnosis and the frequency of NTRK fusions in different cancer types.

The NGS-based approach could represent the ideal method of NTRK fusion detection, especially in patients with advanced disease and few available amounts of biomaterial. The NGS test is recommended in high-grade gliomas, especially in young children, since TRK IHC test has not been validated in this cancer type, which can show physiologic expression of TRK. Unfortunately, the NGS assay is a very expensive technique. Since several variables affect the cost of NGS, namely the library preparation, selection strategy (PCR or capture), sequencer used, bioinformatic analyses, and biological validation, the cost of the test is not easily predictable [71,72].

Recently, an overview of the total and individual component costs of various laboratories has been reported, showing that the total costs of the NGS assay ranged from 376 € to 968 € per oncologic patient [72].

In the detection of TRK fusions, the innovative NGS approach concurrently analyzing DNA and RNA could definitively overcome any limit; however, an increase in costs must be considered. The total NGS cost is on average 607 € per patient, compared to a mean cost of 100 € for Pan-TRK IHC, 100 € for RT-PCR, and a mean of 300 € for FISH analysis, since three different FISH tests of NTRK1, 2, 3 must be performed.

According to the economic feasibility in the clinical practice, the NGS test could be used for the detection of NTRK fusions in all cases with a negative result using IHC, FISH, or RT-PCR as screening assays, in order to detect cases of potential false negatives.

In infantile fibrosarcoma and congenital mesoblastic nephroma, which have a high frequency of ETV6-NTRK3 fusion, FISH or RT-PCR could represent an easy and inexpensive technical approach, with only rare negative cases to be confirmed with NGS.

In all other tumors not frequently associated with ETV6-NTRK3, pan-TRK IHC could be the screening method, since in one assay, it is able to detect indiscriminately all TRK fusions; FISH or RT-PCR tests could be used to confirm IHC-positive cases while only IHC-negative cases should be tested with NGS. In the near future, multidisciplinary teams’ experience in clinical practice will improve this diagnostic flow chart for the identification of NTRK fusions.

## 7. Expert Opinion

The growing interest in NTRK oncogenic aberrations in the latest years has its rationale in the recent development of several selective and non-selective Trk inhibitors whose activity and tolerability are being tested in ongoing clinical trials with encouraging results. Among the most promising agents, entrectinib is a multi-kinase inhibitor that targets TrkA, TrkB, TrkC, ROS1, and ALK1, while larotrectinib is a selective inhibitor of Trk receptors. They are orally available, can cross the brain–blood barrier, and have shown remarkable efficacy and acceptable toxicity profiles in the treatment of metastatic or locally advanced NTRK-fusion malignancies, independently of the histotype, fusion type, and patient age [13,67]. For these reasons, in 2019, larotrectinib and entrectinib were granted accelerated approval by the FDA for adult and pediatric patients with solid tumors harboring NTRK gene fusions. It is clear that the treatment of patients with NTRK fusion-positive cancers with a first-generation NTRK inhibitor achieves high response rates irrespective of the tumor histology, age, or fusion type, and has a good safety profile. Recently, NTRK1/2/3 fusion genes in some oncological settings seem to be associated to specific morphological/molecular profiles, opening the possibility of multidrug therapies. For example, NTRK1/2/3 fusion genes have also been reported as being significantly more frequently found in microsatellite instability (MSI)-high cancers in the context of colorectal carcinoma patients [53].

Several other multikinase inhibitors, which target TRK proteins, are also in clinical development for patients with acquired resistance to first-generation inhibitors. Although available clinical evidence suggests an undoubted role for NTRK inhibitors in selected solid tumors, there are some issues associated with their use, which should be considered, including the approval route for agnostic therapies is to date not well defined, diagnostic procedures are costly, and cost–benefit and quality of life data are lacking.

The final consideration should be dedicated to the definition of cancer patients that should be directed to expensive NTRK fusion gene diagnosis, considering the effective benefits obtained by the specific inhibitors. At present, systematic analyses of large cohorts of metastatic cancer patients for the presence of NTRK1/2/3 fusion genes across cancer types are yet to be carried out. Waiting for possible stringent clinical and pathological criteria of cancer patients harboring NTRK1/2/3 fusion genes, some guidelines, and in particular those by the National Comprehensive Cancer Network (NCCN) on non-small-cell lung cancer, have already included a recommendation for NTRK gene fusion testing in all patients with metastatic disease [73].

In conclusion, with a view to precision medicine and tailored treatment, the cancer population needing to be tested for NTRK fusion genes diagnosis should be represented by any cancers, also in the metastatic stage, as it has mainly been proven a wild type for other targetable gene alteration. Finally, these indications should be followed for young cancer patients.

## Figures and Tables

**Table 1:** Incidence groups of NTRK rearrangements in adult and pediatric tumors.

	Adult Tumors	Pediatric Tumors
<5%	Lung, colorectal, pancreatic, appendiceal cancer, cholangiocarcinoma, melanoma, glioma and several sarcoma histotypes	Glioma and several sarcoma histotypes
5–75%	Thyroid and Gastrointestinal Stromal Tumors (GIST)	Thyroid, Spitzoid tumors and congenital mesoblastic nephroma
>75%	Secretory breast carcinoma and secretory salivary gland tumors	Infantile fibrosarcoma and breast secretory carcinoma
